# Prostaglandin 2α Promotes Autophagy and Mitochondrial Energy Production in Fish Hepatocytes

**DOI:** 10.3390/cells11121870

**Published:** 2022-06-09

**Authors:** Jingjing Tian, Yihui Du, Ermeng Yu, Caixia Lei, Yun Xia, Peng Jiang, Hongyan Li, Kai Zhang, Zhifei Li, Wangbao Gong, Jun Xie, Guangjun Wang

**Affiliations:** 1 Key Laboratory of Aquatic Animal Immune Technology of Guangdong Province, Pearl River Fisheries Research Institute, Chinese Academy of Fishery Sciences, Guangzhou 510380, China; tianjj@prfri.ac.cn (J.T.); duyihui2021@163.com (Y.D.); yem@prfri.ac.cn (E.Y.); leicaixia0703@sina.com (C.L.); yx@prfri.ac.cn (Y.X.); jiangpeng@prfri.ac.cn (P.J.); lihongyan@prfri.ac.cn (H.L.); zk@prfri.ac.cn (K.Z.); lzf@prfri.ac.cn (Z.L.); gwb@prfri.ac.cn (W.G.); 2 Key Laboratory of Tropical and Subtropical Fishery Resource Application and Cultivation, Pearl River Fisheries Research Institute, Chinese Academy of Fishery Sciences, Guangzhou 510380, China

**Keywords:** arachidonic acid, ATP, cyclooxygenase, eicosanoids, lipid droplets, lipolysis, lipophagy, mitochondria, PGF2α

## Abstract

Fatty liver, characterized by excessive lipid droplet (LD) accumulation in hepatocytes, is a common physiological condition in humans and aquaculture species. Lipid mobilization is an important strategy for modulating the number and size of cellular LDs. Cyclooxygenase (COX)-mediated arachidonic acid derivatives are known to improve lipid catabolism in fish; however, the specific derivatives remain unknown. In the present study, we showed that serum starvation induced LD degradation via autophagy, lipolysis, and mitochondrial energy production in zebrafish hepatocytes, accompanied by activation of the COX pathway. The cellular concentration of PGF2α, but not other prostaglandins, was significantly increased. Administration of a COX inhibitor or interference with PGF2α synthase abolished serum deprivation-induced LD suppression, LD–lysosome colocalization, and expression of autophagic genes. Additionally, exogenous PGF2α suppressed the accumulation of LDs, promoted the accumulation of lysosomes with LD and the autophagy marker protein LC3A/B, and augmented the expression of autophagic genes. Moreover, PGF2α enhanced mitochondrial accumulation and ATP production, and increased the transcript levels of β-oxidation- and mitochondrial respiratory chain-related genes. Collectively, these findings demonstrate that the COX pathway is implicated in lipid degradation induced by energy deprivation, and that PGF2α is a key molecule triggering autophagy, lipolysis, and mitochondrial development in zebrafish hepatocytes.

## 1. Introduction

With rapid industrialization, obesity and fatty liver disease have become non-communicable diseases that threaten modern societies worldwide [1]. Coincidentally, in the aquaculture industry, to pursue optimal growth and maximize profit, high-energy commercial diets are widely supplied to fish such as grass carp [2,3], tilapia [4], and large-mouth bass [5], but these diets also induce excessive fat accumulation in the liver and mesentery. Furthermore, these impacts not only diminish feed assimilation efficiency, but also negatively affect the health and market value of fish [6]. Fatty liver is characterized by the accumulation of lipid droplets (LDs), which are mainly composed of triglycerides and sterol esters, in hepatocytes [7]. LD degradation is an important process for regulating the size and number of LDs [8]; thus, it is a promising therapeutic approach. Moreover, increased fat utilization is an important strategy in aquaculture because it spares proteins, which are expensive feed components [9]. Several cytoplasmic lipases, such as adipose tissue lipase (ATGL) and hormone-sensitive lipase (HSL), are involved in triglyceride lipolysis and release free fatty acids from triglycerides in LDs [10]. These enzymes are also detected in fish, and their functions and regulatory mechanisms appear to be evolutionarily conserved [11,12,13]. Concurrently, autophagy is a response to LD degradation in the liver of mice and fish. Specifically, autophagy is mediated by the formation of double-membrane autophagosomes at the surface of LDs to transport triglycerides to lysosomes for degradation; the entire process is termed lipophagy [14,15,16]. ATGL has been proposed as a direct regulator of autophagy/lipophagy in mice [17]. Free fatty acids released through lipolysis or autophagy/lipophagy are transported to the mitochondria for β-oxidation by a series of enzymes to produce acetyl-CoA as the end product [18]. Acetyl-CoA then enters the tricarboxylic acid cycle to form NADPH and FADH2, which are absorbed by the mitochondrial respiratory chain, through which five enzymatic complexes produce ATP [19,20].

Arachidonic acid (ARA; C20:4n-6) is a major n-6 long-chain polyunsaturated fatty acid that is primarily esterized at the sn-2 position of phospholipids in the cell membrane [21]. ARA is liberated from the cell membrane via phospholipase A2 (PLA2) in response to cellular activation by cytokines, growth factors, and mechanical trauma [22]. Free ARA serves as a precursor for one of the most important groups of bioactive lipid mediators, eicosanoids. There are three major enzymatic routes of eicosanoid synthesis: (i) the cyclooxygenase (COX) pathway, which leads to the formation of prostaglandins (PGs) and thromboxane; (ii) the lipoxygenase pathway, which results in the formation of leukotrienes and hydroxyeicosatetraenoic acid with different regioisomers; and (iii) the cytochrome P 450 pathway, which generates hydroxyeicosatetraenoic acid and epoxyeicosatetraenoic acid [23,24]. These eicosanoids are involved in numerous complex homeostatic and physiological processes in an autocrine or paracrine manner [24].

ARA and its metabolites may play pivotal roles in fish lipid catabolism. Previously, we showed that dietary ARA increases ATGL transcription and serum non-esterified fatty acid levels in freshwater grass carp. Moreover, acetylsalicylic acid, a COX inhibitor, abolishes the lipolysis-promoting function of ARA, suggesting that increased cellular ARA levels facilitate lipolysis and that COX-mediated metabolites are involved in the regulation of this process [25,26,27]. However, the specific metabolites that promote lipolysis have not yet been elucidated. Moreover, whether ARA-derived metabolites promote autophagy in fish remains unclear. Interestingly, studies in mammals have revealed the diverse regulatory roles of COX-mediated metabolites in lipid accumulation. For instance, PGI2 acts as a potent promoter of lipolysis in adipocytes [28], whereas 15-deoxy-Δ^12,14^-PGJ2 is an endogenous ligand of the adipogenic protein PPARγ [29]. Meanwhile, it remains controversial whether PGE2 inhibits or is not involved under the basal lipolysis status but promotes lipolysis under the activated lipolysis status [28,30,31]. However, to date, no study has explored the function of PGF2α or TXA2 in the regulation of lipid catabolism in mammals. Nonetheless, a previous study showed that starvation-induced autophagy is accompanied by increased protein expression of enzymes involved in PGF2α synthesis in grass carp adipose tissue [32]. Notably, in mammals, PGF2α induces autophagy during luteolysis [33,34].

In the present study, we used a zebrafish liver cell line, an academically accepted model for studying LD regulation, to preliminarily explore the dynamics of ARA-derived COX metabolites in response to autophagy and lipolysis activation and to elucidate the key molecules regulating lipid catabolism and oxidation in fish hepatocytes.

## 2. Materials and Methods

### 2.1. Cell Culture and Treatments

The zebrafish liver (ZFL) cell line was purchased from American Type Culture Collection (ATCC, Manassas, VA, USA). Cells were cultured in a mixed medium (50% L-15, 35% DMEM HG, and 15% Ham’s F12; Gibco, Thermo Fisher Scientific, Waltham, MA, USA) supplemented with 0.15 g/L sodium bicarbonate (Sigma-Aldrich, St. Louis, MO, USA), 15 mM HEPES (Sigma-Aldrich), 0.01 mg/mL bovine insulin (Sigma-Aldrich), 50 ng/mL epidermal growth factor (Sigma-Aldrich), 5% fetal bovine serum (Gibco), and 0.5% trout serum (Caisson Labs, Smithfield, UT, USA). The cells were incubated at 28 °C in a 100% air atmosphere. The medium was replaced every 2 days.

LD-containing cells were prepared by adding 400 μM lipid mixture (200 μM oleic acid, 100 μM linoleic acid, and 100 μM linolenic acid; coated with bovine serum albumin; Sigma-Aldrich) for 24 h. LD-containing cells were directly incubated with PGF2α and PGE2 (MedChemExpress, Monmouth Junction, NJ, USA; dissolved in dimethyl sulfoxide). Before treatment, cytotoxicity of the molecules was assessed using a 3-(4,5-dimethyl-2-thiazolyl)-2,5-diphenyl-2H-tetrazolium bromide assay.

### 2.2. RNA Interference and Inhibitor Treatment

The cells were not transfected with siRNA or treated with the inhibitor until they reached 75% confluency. For RNA interference, the cells were transfected with specific siRNA sequences against ATG7 (sense: 5′-GGGACCCAGAAGUGUUAAUTT-3′; antisense: 5′-AUUAACACUUCUGGGUCCCTT-3′), ATGL (sense: 5′-CCGGACACCAACAGGACAUTT-3′; antisense: 5′-AUGUCCUGUUGGUGUCCGGTT-3′), and PTGFS (sense: 5′-GGGUGACAUCUACAUUGAUTT-3′; antisense: 5′-AUCAAUGUAGAUGUCACCCTT-3′), or nontargeting siRNA (negative control; 5′-UUCUCCGAACGUGUCACGUTT-3′), for 24 h with a transfection reagent (Invitrogen, Thermo Fisher Scientific, Waltham, MA, USA). The siRNAs were designed and synthesized by GenePharma (Shanghai, China). The gene knockdown efficiency was 74.12% for ATG7, 54.38% for ATGL, and 45.89% for PTGFS (Figures S1a and S2a). For inhibitor treatment, cells were incubated with ibuprofen (MedChemExpress), chloroquine (CQ; 5 or 50 μM; MedChemExpress), or non-inhibitor medium for 24 h, followed by treatment with a lipid mixture to obtain LD-containing cells and other treatments.

### 2.3. LD Staining

ZFL cells were seeded at 1.2 × 10^6^/well in six-well plates for 24 h, with three replicates per assay. After treatment, the cells were washed twice with phosphate-buffered saline (PBS) and fixed in 10% formalin for 30 min. After rinsing twice with PBS, the cells were stained with BODIPY solution (10 μg/mL; Invitrogen, Thermo Fisher Scientific, Waltham, MA, USA) for 30 min. After washing the cells three times with PBS to remove the BODIPY solution, the nuclei were stained with DAPI solution (10 μg/mL; Invitrogen, Thermo Fisher Scientific) for 10 min. Finally, after removing the DAPI solution and washing with PBS, the cells were observed under an inverted fluorescence microscope with a ×40 objective (Nikon TS2, Tokyo, Japan). Three nonoverlapping images were obtained for each treatment group. Eighteen cells (six cells per image) in each treatment were randomly selected, and the particle number was quantified using ImageJ software (National Institutes of Health, Bethesda, MD, USA). In addition, the relative puncta of BODIPY fluorescence values and cell numbers (in each image) were quantified using ImageJ software (1.53q; National Institutes of Health, Bethesda, MD, USA).

### 2.4. Immunofluorescence Staining

For immunofluorescence microscopy, ZFL cells were seeded in six-well plates at 1.2 × 10^6^/well for 24 h prior to treatment, with three replicates per assay. The cells were fixed in 4% paraformaldehyde for 30 min and then washed three times with PBS. The cells were then incubated with Triton X-100 (0.5%) for 10 min and washed with PBS. After blocking with 5% bovine serum albumin for 30 min, the cells were incubated with the primary antibody (LC3A/B; Cell Signaling Technology, Danvers, MA, USA; dissolved in fetal bovine serum) overnight (4 °C). After washing with PBS, the cells were incubated with a secondary antibody conjugated to Alexa Fluor 488 (Cell Signaling Technology) for 2 h. The cell LDs and nuclei were then stained. The cells were observed and imaged under an inverted fluorescence microscope with a ×40 objective (Nikon TS2). The relative puncta of fluorescence values and cell numbers (in each image) were quantified using ImageJ software.

### 2.5. PG Analysis

For the PG test, cells were seeded at a density of 1.0 × 10^6^/well in 25 cm plastic bottles for 24 h prior to treatment. Six replicates were used for each treatment. At harvest, the cells were digested with 0.25% trypsin–EDTA, washed twice with PBS, and suspended in 100 μL PBS, and the cell medium was collected. The cells were then subjected to three freeze–thaw cycles (−80 °C) and centrifuged for 10 min at 5000× *g* at 4 °C. The supernatant was then transferred to a fresh Eppendorf tube for further analysis. Major PGs, including 15d-PGJ2, PGE2, PGF2α, and PGI2, were quantified using an enzyme-linked immunosorbent assay kit (MEIMIAN, Wuhan, China) according to the manufacturer’s protocol, using a microtiter plate spectrophotometer (Multiskan MK3, Thermo Labsystems, Philadelphia, PA, USA) at an absorbance of 450 nm. All metabolite assays were performed in duplicates. The results are expressed as nanograms of PGs per microgram of protein (for cells) or milliliter (for medium).

### 2.6. Lysosomal and Mitochondrial Staining

ZFL cells were seeded in six-well plates at 1.2 × 10^6^/well for 24 h prior to treatment, with three replicates per assay. For lysosomal staining, growing cells were incubated with 20 nM LysoTracker Red DND-99 (Invitrogen, Thermo Fisher Scientific) for 30 min at 28 °C before fixation. For mitochondrial staining, growing cells were incubated with 100 nM MitoTracker Red FM (Invitrogen, Thermo Fisher Scientific, Waltham, MA, USA) for 30 min at 28 °C before fixation. The cell LDs and nuclei were stained and captured under an inverted fluorescence microscope with a ×40 objective (Nikon TS2). The relative puncta of fluorescence values and cell numbers (in each image) were quantified using ImageJ software. The Mander’s overlap coefficient of LDs and lysosome puncta was measured using the Coloc 2 plug-in.

### 2.7. Triglyceride (TG), Free Fatty Acid, and Glycerol Content

For TG assays, ZFL cells were seeded in six-well plates at 1.2 × 10^6^/well for 24 h prior to treatment, with four replicates per assay, lysed, and collected. TG assays were performed using an enzymatic kit (Applygen, Beijing, China). The cell medium was collected after treatment and measured using free fatty acid and glycerol assay kits (Jiancheng Biotech Co., Nanjing, China).

### 2.8. ATP Determination

To measure ATP generation, ZFL cells were seeded in six-well plates at 1.2 × 10^6^/well for 24 h prior to treatment, with six replicates per treatment. A commercial kit (GENMED, Shanghai, China) was used to normalize the total protein concentration, according to the manufacturer’s instructions.

### 2.9. Real-Time Quantitative Reverse Transcription-PCR (qRT-PCR)

For qRT-PCR, ZFL cells were seeded in six-well plates at 1.2 × 10^6^/well. Each treatment was replicated three times. After treatment, the cells were washed twice with PBS, and 1 mL TRIzol reagent (Life Technologies Inc., Thermo Fisher Scientific, Waltham, MA, USA) was added to extract total RNA, according to the manufacturer’s instructions. RNA integrity was assessed using agarose gel electrophoresis and an Implen NanoPhotometer (Implen Inc., Westlake Village, CA, USA). After removing DNA from the total RNA, cDNA was synthesized using the PrimeScript^®^ RT Reagent Kit (TaKaRa, Otsu, Japan). qRT-PCR assays were performed in triplicate using the LightCycler^®^ 96 real-time PCR machine (Roche, Basel, Switzerland) in a 20 μL reaction system containing 2.0 μL primers (2.5 μM), 2.0 μL diluted first-strand cDNA, 10 μL 2× Power SYBR™ Green PCR Master Mix (Thermo Fisher Scientific), and 6.0 μL sterilized double-distilled water. The cycling conditions were as follows: 95 °C for 5 min, followed by 40 cycles of 95 °C for 15 s, and finally, 60 °C for 1 min. After PCR, melting curves were analyzed over a range of 72–95 °C (at 1 °C/20 s steps) to confirm product singularity. Relative gene expression levels were determined using the comparative CT method (2^−ΔΔCt^), as described previously [35,36].

### 2.10. Mitochondrial DNA (mtDNA) Content Assay

For the mtDNA assay, ZFL cells were seeded in six-well plates at 1.2 × 10^6^/well. Each treatment was replicated four times. To measure mtDNA, total DNA was extracted using a DNA extraction kit (QIA GEN, Beijing, China), following the manufacturer’s instructions. In the present study, the copy number of mitochondrial cytochrome b (mt-cyb) was considered the copy number of mtDNA. β-actin was used as the internal reference. qRT-PCR was performed as described previously (Section 2.9).

### 2.11. Statistical Analysis

All data are expressed as the mean ± standard deviation. One-way analysis of variance followed by Bonferroni’s post hoc test was used to compare the differences between experimental treatments. Differences between the non-starved and starved cells were determined using an independent sample *t*-test. All analyses were performed using PASW Statistics 18 software (SPSS, Chicago, IL, USA).

## 3. Results

### 3.1. Serum Starvation Induces Autophagy, Lipid Depletion, and Mitochondrial Energy Production in ZFL Cells

**Figure 1:**
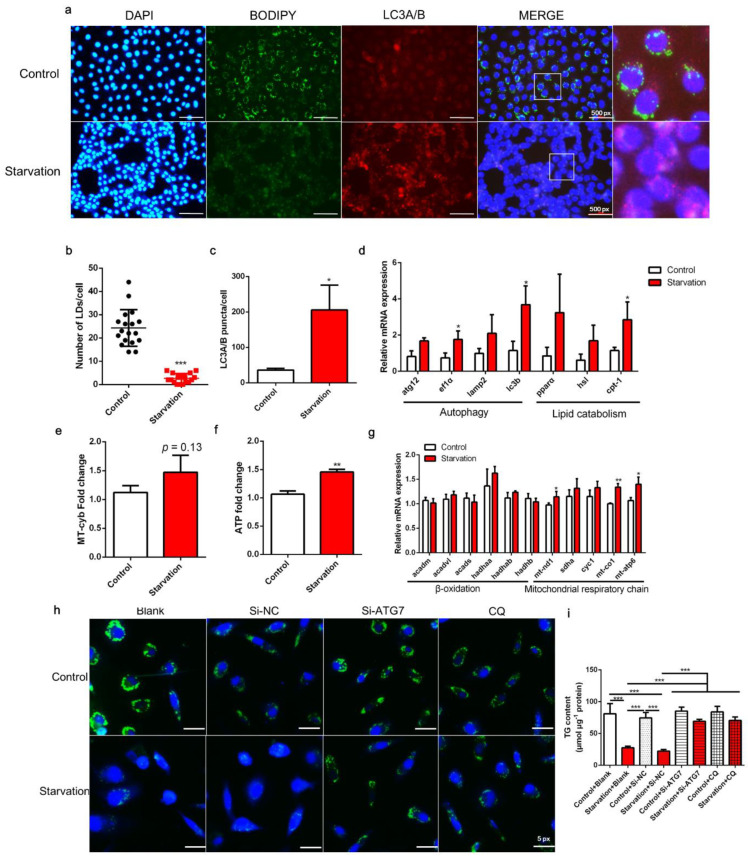
Effects of serum starvation on lipid droplet (LD) accumulation, autophagy, and mitochondrial energy production in zebrafish liver cells. Cells were incubated with either normal medium or serum-free medium for 24 h (**a**–**g**). (**a**) LDs were stained with BODIPY (green); nuclei were stained with DAPI (blue); and LC3A/B was stained with a specific immunofluorescent antibody (red). (**b**) LDs in 18 cells (6 cells in each image) were quantified using ImageJ. (**c**) Relative fluorescence puncta of LC3A/B per cell were quantified using ImageJ (*n* = 3). (**d**) Relative transcript expression of autophagy- and lipid catabolism-related genes (*n* = 3). (**e**) Mitochondrial copy number (*n* = 4). (**f**) ATP level (*n* = 6). (**g**) Relative transcript expression of β-oxidation- and mitochondrial respiratory chain-related genes (*n* = 3). (**h**,**i**) Cells were pre-treated with siRNA against negative control (NC), ATG7, or CQ and then serum-starved for 24 h. LDs were stained with BODIPY, and the TG content was measured (*n* = 4). atg12, autophagy-related 12; ef1α, elongation factor 1 α; lamp2, lysosomal-associated membrane protein 2; lc3b, microtubule-associated protein 1 light chain 3b; atgl, adipose tissue lipase; hsl, hormone-sensitive lipase; cpt-1, carnitine palmitoyltransferase 1; acadm, acyl-CoA dehydrogenase medium chain; acadvl, acyl-CoA dehydrogenase very long chain; acads, acyl-CoA dehydrogenase short chain; hadh, hydroxyacyl-CoA dehydrogenase trifunctional multienzyme complex; mt-nd1, NADH dehydrogenase 1, mitochondrial; sdha: succinate dehydrogenase complex, subunit A; cyc1: cytochrome c-1; mt-co1: mitochondrial cytochrome c oxidase; mt-atp: mitochondrial ATP synthase. Statistical significance is denoted with asterisks as follows: * *p* < 0.05; ** *p* < 0.01; *** *p* < 0.001.

The number of LDs (green fluorescent spots) in serum starvation-treated ZFL cells was clearly decreased compared with that in control cells (*p* < 0.05; Figure 1a,b). Moreover, the expression of the autophagy marker protein LC3A/B (red fluorescent spots) increased after starvation treatment (*p* < 0.05; Figure 1a,c). Meanwhile, the transcript expression of the autophagy marker genes *atg12*, *ef1α*, *lamp2*, and *lc3b* was increased in serum-starved cells, with the expression of *ef1α* and *lc3b* being significantly different from that of the control (*p* < 0.05; Figure 1d). Likewise, expression of the lipid catabolic genes *pparα*, *hsl*, and *cpt-1* was increased in starved cells, with *cpt-1* expression showing a significant difference compared to the control (*p* < 0.05; Figure 1d). Furthermore, serum starvation increased mitochondrial copy number (*p* = 0.13; Figure 1e) and significantly increased ATP production (*p* < 0.05; Figure 1f). Several genes related to the mitochondrial respiratory chain, such as *mt-nd1*, *mt-co1*, and *mt-apt*, were significantly upregulated in response to starvation (*p* < 0.05; Figure 1g). These results demonstrate that serum starvation induces lipid degradation and autophagy, and reduces LD accumulation in ZFL cells. Furthermore, we administered RNA interference of ATG7, a key gene in autophagy, with an interference efficiency of 74.12% (Figure S1a), and an autophagy inhibitor, CQ, which has been demonstrated to effectively inhibit autophagy of ZFL [16], to block autophagic flux. Results showed that autophagy inhibition through ATG7 interference or CQ administration rescued lipid degradation induced by starvation to a certain extent (*p* < 0.05; Figure 1h,i), suggesting that autophagy contributes to serum starvation-induced LD degradation in ZFL cells.

### 3.2. Serum Starvation Induces COX Activity in ZFL Cells

**Figure 2:**
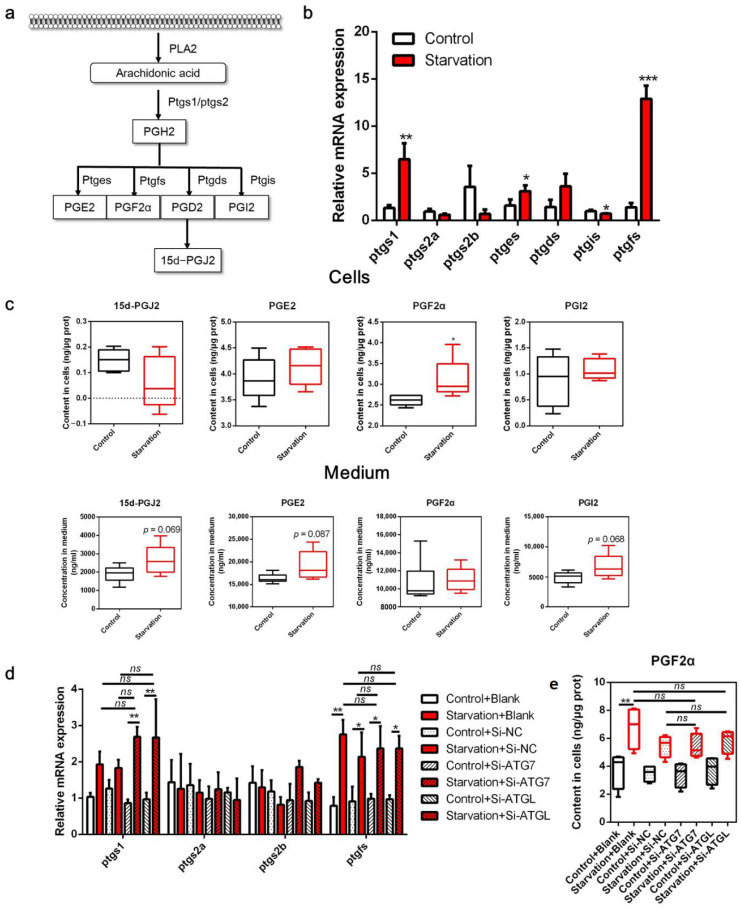
Effects of serum starvation on cyclooxygenase (COX) metabolism in zebrafish liver cells. (**a**) Schematic diagram of the COX pathway in arachidonic acid (ARA) metabolism. (**b**,**c**) Cells were incubated with either normal medium or serum-free medium for 24 h, and the relative transcript expression of COX metabolism-related genes (*n* = 3) and the concentration of ARA-derived COX metabolites in the cells and medium were measured (*n* = 5). (**d**,**e**) Cells were pre-treated with NC, ATG7, and ATGL siRNA and then serum-starved for 24 h, and the relative mRNA expression of COX metabolism-related genes (*n* = 3) and the concentration of PGF2α in cells (*n* = 6) were measured. Ptgs, prostaglandin-endoperoxide synthase; pgtes, prostaglandin E synthase; ptgds, prostaglandin D2 synthase; ptgis, prostaglandin I2 synthase; ptgfs, prostaglandin F synthase. Statistical significance is denoted with asterisks as follows: * *p* < 0.05; ** *p* < 0.01; *** *p* < 0.001; ns, not significant difference.

In the COX pathway, PTGS converts ARA to PGH2, which is then converted to PGD2, PGE2, PGF2α, and PGI2 by the action of enzymes PTGDS, PTGES, PTGFS, and PGTGIS. Spontaneous PGD2 dehydration resulted in PGJ2 formation (Figure 2a). In the present study, serum starvation significantly increased the transcript levels of ptgs1 (*p* < 0.05, Figure 2b), but not of *ptgs2a* and *ptgs2b* (*p* > 0.05). Moreover, *ptges*, *ptgfs*, and *ptgds* expression was increased in cells treated with serum-free medium, with *ptges* and *ptgfs* expression being significantly different compared to the control (*p* < 0.05). However, *ptgis* transcript expression was downregulated in the starved cells (*p* < 0.05). Accordingly, ARA-derived COX metabolites in the cells and medium were measured (Figure 2c). The PGF2α concentration was significantly increased in serum-starved cells (*p* < 0.05), whereas no obvious differences were noted in the concentrations of other PGs, including 15-dPGJ2, PGE2, and PGI2 (*p* > 0.05). Interestingly, although the concentrations of these four metabolites were not significantly different in the medium, 15-dPGJ2, PGE2, and PGI2 concentrations markedly increased in numerical value (*p* > 0.05). To investigate whether PGF2α is derived from LD degradation induced by serum starvation, we knocked down ATG7 and ATGL using RNA interference. The expression of ATG7 and ATGL (a key gene for lipolysis) was significantly decreased (Figure S1a), and starvation-induced LD depletion was blocked by interfering with these two genes (Figure S1b,c). In addition, starvation-induced release of free fatty acids and glycerol was abrogated by disturbing autophagy and lipolysis (Figure S1d,e), suggesting that autophagy and lipolysis were successfully impaired. Interestingly, abolishing autophagy and lipolysis through ATG7 and ATGL interference neither eliminated *ptgs1* and *ptgfs* upregulation nor elevated PGF2α concentration induced by serum starvation (Figure 2d,e). These results demonstrate that serum deprivation triggers the COX pathway in ZFL cells in a non-LD degradation manner.

### 3.3. COX Pathway Is Implicated in Serum Starvation-Induced Autophagy and LD Degradation in ZFL Cells

**Figure 3:**
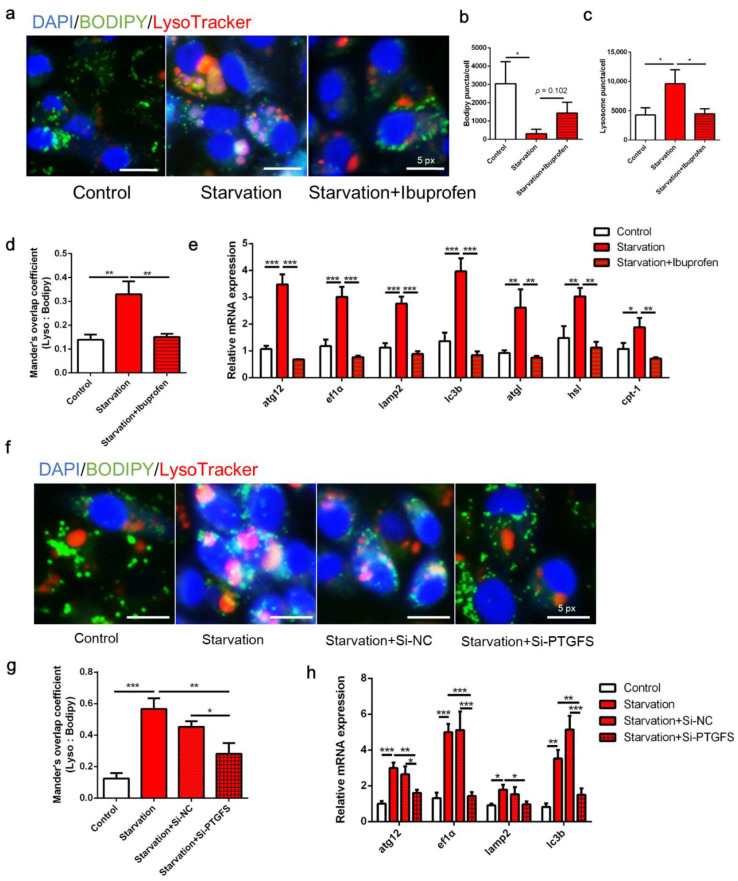
Effects of cyclooxygenase (COX) pathway inhibition on serum starvation-induced autophagy and lipid droplet (LD) degradation in zebrafish liver cells. (**a**–**e**) Cells were administered the COX inhibitor ibuprofen (10 μM) and then serum-deprived for 24 h. (**f**–**h**) Cells were pre-interfered with Si-NC or Si-PTGFS then treated with serum deprivation for 6 h. LDs were stained with BODIPY (green), nuclei were stained with DAPI (blue), and lysosomes were stained with LysoTracker (red). LD and lysosome puncta per cell as well as Mander’s overlap coefficient were measured using Image J (*n* = 3). Relative expression of autophagy-related genes was tested using qRT-PCR (*n* = 3). atg12, autophagy-related 12; ef1α, elongation factor 1α; lamp2, lysosomal-associated membrane protein 2; lc3b, microtubule-associated protein 1 light chain 3b; atgl adipose tissue lipase; hsl, hormone-sensitive lipase; cpt-1, carnitine palmitoyltransferase 1. Statistical significance is denoted with asterisks as follows: * *p* < 0.05; ** *p* < 0.01; *** *p* < 0.001.

Figure 3 presents the autophagy and lipid catabolism in ZFL cells in response to COX pathway inhibition before serum starvation. First, serum deprivation eliminated LDs in ZFL cells, whereas the administration of the COX inhibitor ibuprofen recovered lipid accumulation (*p* < 0.05; Figure 3a,b). From the perspective of autophagy, serum starvation increased the puncta of lysosomes in the cells, whereas the number of lysosomes decreased following ibuprofen treatment (*p* < 0.05; Figure 3a,c). Moreover, ibuprofen administration significantly attenuated the colocalization of LDs and lysosomes induced by serum starvation (*p* < 0.05; Figure 3a,d). Serum starvation significantly increased the expression of autophagy- and lipolysis-related genes, including *atg12*, *ef1α*, *lamp2*, *lc3b*, *atgl*, *hsl*, and *cpt-1*, whereas all of these genes were attenuated by ibuprofen treatment (Figure 3e, *p* < 0.05). Second, knockdown of PTGFS by RNA interference decreased the expression of PTGFS by 45.89% and reduced the production of PGF2α (Figure S2a,b), which abolished lysosome accumulation and decreased LD colocalization with lysosomes induced by starvation (Figure 3f,g, *p* < 0.05). Furthermore, PTGFS knockdown significantly attenuated the starvation-induced expression of the autophagy-related genes *atg12*, *ef1α*, *lamp2,* and *lc3b* (Figure 3h, *p* < 0.05). Overall, these results indicate that COX and PTGFS inhibition can restore serum starvation-induced lipid degradation and autophagy.

### 3.4. PGF2α Promotes Autophagy in ZFL Cells

**Figure 4:**
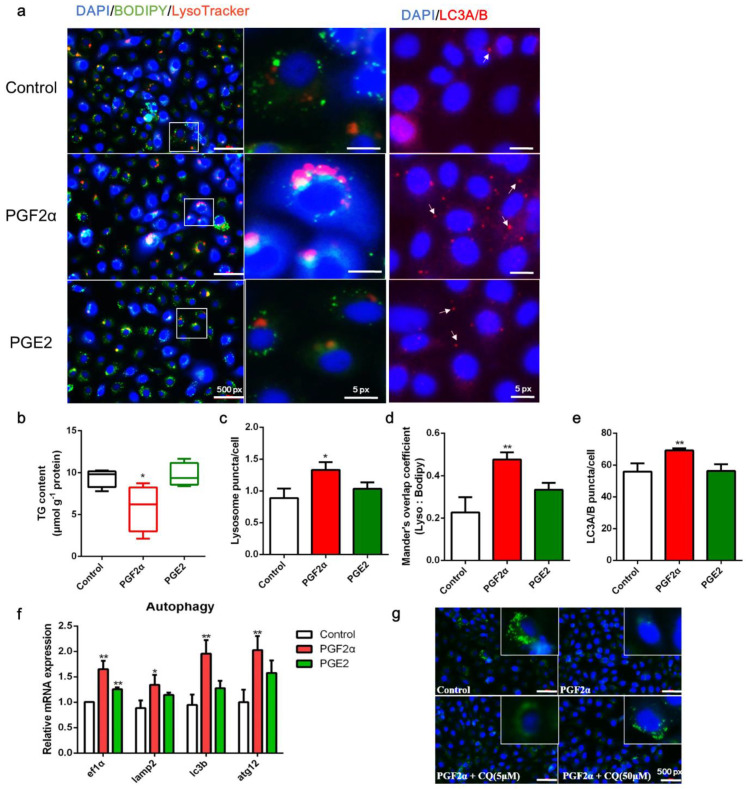
Effects of PGF2α and PGE2 on autophagy and lipid accumulation in zebrafish liver cells. Cells were incubated with or without PGF2α (10 μM) and PGE2 (10 μM) for 24 h. (**a**) Lipid droplets (LDs) were stained with BODIPY (green), nuclei were stained with DAPI (blue), lysosomes were stained with LysoTracker (red), and LC3A/B was stained with a specific immunofluorescent antibody (red). (**b**–**e**) Triglyceride (TG) content (*n* = 4), relative fluorescence of lysosome puncta (*n* = 3) and LC3A/B (*n* = 3), and Mander’s overlap coefficient of lysosome/BODIPY (*n* = 3) in each image were quantified using ImageJ software (*n* = 4). (**f**) Relative transcript expression of autophagy-related genes (*n* = 3). (**g**) Cells were pre-incubated with autophagy inhibitor CQ, then treated with PGF2α for 24 h; the LDs and nuclei were stained with BODIPY and DAPI, respectively. ATG12, autophagy-related 12; EF1α, elongation factor 1 α; LAMP2, lysosomal-associated membrane protein 2; LC3b, microtubule-associated protein 1 light chain 3b. Statistical significance is denoted with asterisks as follows: * *p* < 0.05; ** *p* < 0.01.

To determine whether PGF2α is involved in the regulation of autophagy, LD-containing ZFL cells were treated with either PGF2α or PGE2. Compared with the control, PGF2α decreased LD accumulation and TG content but increased lysosome content, LD colocalization with lysosomes, and LC3A/B expression in ZFL cells (*p* < 0.05, Figure 4a–e). In addition, PGF2α significantly increased the transcript expression of autophagy-related genes *ef1α*, *lamp2*, *lc3b*, and *atg12* (*p* < 0.05, Figure 4f). Meanwhile, PGE2 showed a weaker ability to induce autophagy than PGF2α (Figure 4a–e). Finally, inhibition of autophagy by CQ administration rescued the depletion of LDs induced by PGF2α (Figure 4g). Overall, these results suggest that PGF2α promotes autophagy in ZFL cells, which may be a key step in LD suppression.

### 3.5. PGF2α Promotes Mitochondrial Energy Production in ZFL Cells

**Figure 5:**
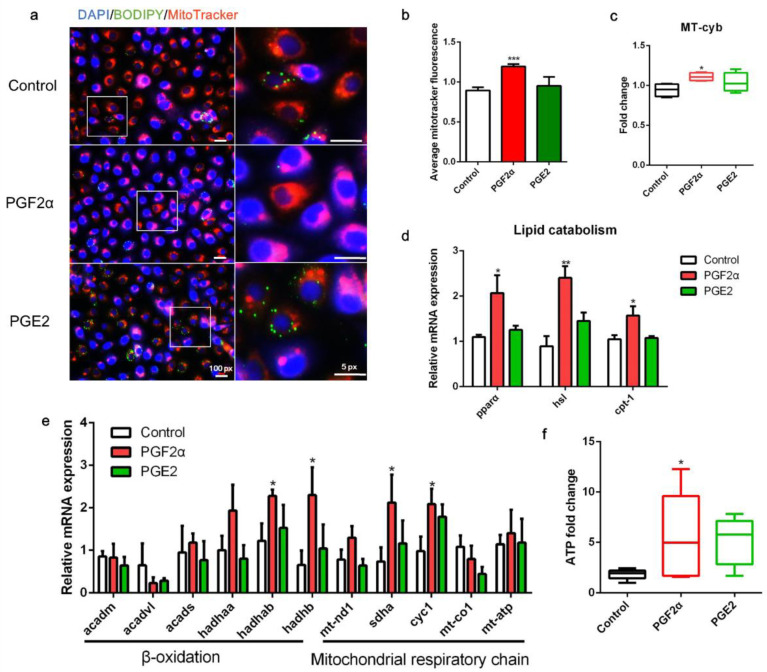
Effects of PGF2α and PGE2 on mitochondrial function in zebrafish liver cells. Cells were incubated with or without PGF2α (10 μM) and PGE2 (10 μM) for 24 h. (**a**) Lipid droplets were stained with BODIPY (green), nuclei were stained with DAPI (blue), and mitochondria were stained with MitoTracker (red). (**b**) Average MitoTracker fluorescence per cell (*n* = 3). (**c**) Mitochondrial copy number (*n* = 4). (**d**) Relative transcript expression of lipid catabolism-related genes (*n* = 3). (**e**) Relative transcript expression of β-oxidation- and mitochondrial respiratory chain-related genes (*n* = 3). (**f**) ATP levels (*n* = 6). Statistical significance is denoted with asterisks as follows: * *p* < 0.05; ** *p* < 0.01; *** *p* < 0.001.

As shown in Figure 5, following PGF2α treatment, the mitochondrial content in the cells increased (Figure 5a,b). Furthermore, the copy number of mtDNA was significantly higher in PGF2α-treated cells than in control cells (*p* < 0.05; Figure 5c). PGF2α significantly promoted the transcript expression of lipid catabolism-related genes, including *pparα*, *hsl*, and *cpt-1 (p* < 0.05, Figure 5d). Interestingly, PGF2α treatment significantly increased the transcript expression of mitochondrial β-oxidation-related trifunctional multienzymes *hadhab* and *hadhb*, and mitochondrial respiratory chain-related genes *sdha* and *cyc1* (*p* < 0.05, Figure 5e). Finally, PGF2α treatment significantly increased ATP production in ZFL cells (*p* < 0.05; Figure 5f). However, PGE2 treatment did not significantly affect mitochondrial content, LD degradation, or ATP production in ZFL cells (*p* > 0.05, Figure 5a–f). Collectively, these results indicate that PGF2α promotes mitochondrial energy production in ZFL cells.

## 4. Discussion

Autophagy/lipophagy and lipolysis are biological processes conserved throughout the course of evolution from yeast to humans [37]. However, the complex regulatory network of autophagy/lipophagy is far from being well-described, particularly in fish [38,39]. In addition, the involvement of cell-metabolized molecules, particularly the molecular forms of fatty acid derivatives, in this process has rarely been studied. In the present study, we demonstrated that the levels of an ARA-derived eicosanoid from the COX pathway, PGF2α, increased in ZFL cells, along with an increase in lipid catabolism, autophagy, and lipolysis. Furthermore, inhibition of the COX pathway and PGF2α synthase rescued this starvation-induced LD degradation, and autophagy and mitochondrial oxidative function were augmented in fish hepatocytes incubated with PGF2α, but not PGE2. To the best of our knowledge, the present study provides the first evidence that ARA-derived eicosanoids may be regulated during cell autophagy, and that PGF2α may be a key molecule involved in lipid catabolism and fatty acid oxidation in fish hepatocytes.

LDs are dynamically regulated cellular fat particles that are mainly involved in energy storage. As such, sufficient energy availability in the environment results in the formation of LDs, whereas energy utilization by organisms leads to the breakdown of LDs through lipolysis or/and autophagy/lipophagy [40]. Liberated fatty acids are transported to the mitochondria for β-oxidation, the tricarboxylic acid cycle, and the mitochondrial respiratory chain, which finally produce ATP [40,41]. Our finding that serum starvation reduced LD levels is consistent with previous reports in mouse hepatocytes and ZFL cells [14,16,41], and this reduction is attributed to increased lipid catabolism and ATP production. Indeed, in the present study, the expression of autophagic and lipolytic marker genes or proteins, the level of ATP, and the expression of mitochondrial genes were increased (Figure 1), demonstrating that serum deprivation is a universal measure for studying the mechanism of LD degradation. Furthermore, the observed LD depletion was abolished through autophagy interference, indicating that autophagy is indeed involved in lipid catabolism in response to starvation, consistent with previous reports [16,41]. Overall, our model for studying the mechanism of LD degradation is accurate and can be used in future studies.

Starvation may trigger the activity of cPLA2, which is the key enzyme that hydrolyzes ARA from phospholipids in the cell membrane [42,43]. Moreover, serum starvation increases the expression of COX and promotes the production of related metabolites in mammals [44,45,46]. In the present study, the expression of *ptgs1*, but not *ptgs2a* or *ptgs2b*, increased in serum-deprived cells, suggesting an increased capacity for the conversion of COX-mediated derivatives. Interestingly, *pgts1* is generally acknowledged as a constitutively expressed gene, whereas *pgts2* is an inducible gene in mammals [47]. In the present study, serum starvation upregulated *pgts1* but not *ptgs2*, suggesting contradictory roles for these two paralogs in fish. There are four major ARA-derived PGs: PGJ2, PGE2, PGF2α, and PGI2 [24]. Among the four key enzymes involved in PG synthesis, ptgfs (the enzyme synthesizing PGF2α) was the most highly expressed. Accordingly, PGF2α levels were significantly higher in the cells than in the medium, implying that PGF2α was triggered in response to serum starvation. Interestingly, the levels of other PGs, such as PGE2, PGI2, and 15d-PGJ2, were not increased in the cells, although their levels were increased in the medium. These results indicate the presence of another regulatory mechanism in which PGs other than PGF2α are secreted to the outside cells owing to their paracrine properties [23]; however, the precise mechanism remains to be further addressed. Notably, LD degradation did not appear to be the source of ARA-derived PGF2α production, as suggested by the gene interference experiment. Whether PGF2α originates from the cell membrane, whether its release is the cause, and whether it acts as an intermediate metabolite of lipid degradation remains unknown.

Dietary ARA reduces liver fat in mammals [48,49]. Furthermore, exogenous ARA supplementation reduces lipid accumulation in fish such as grass carp [50], *Synechogobius hasta* [51], and gilthead seabream fingerlings [52]. Increased lipid degradation and decreased lipid synthesis are the major mechanisms underlying the suppression of lipid accumulation in fish, and occur in COX-dependent and COX-independent manners, respectively [25,26,27]. In the present study, treatment with the COX inhibitor ibuprofen abolished the expression of lysosome accumulation-, autophagy-, and lipolysis-related genes induced by nutrition deprivation (Figure 3), and decreased the colocalization of LDs and lysosomes, suggesting that the COX pathway acts upstream of autophagy and may be involved in LD degradation in zebrafish hepatocytes. Interestingly, the knockdown of PTGFS, one of the downstream genes of the COX pathway responsible for PGF2α synthesis [24], attenuated contact between lysosomes and LDs in response to starvation, suggesting that this pathway is important relative to autophagy and lipophagy. Collectively, the activation of the COX–PTGFS pathway may be a steady physiological mechanism in fish in response to nutritional changes in the extracellular environment. However, whether differences in COX paralogs or PG synthases regulate these physiological changes in fish remains unknown.

Previous studies in mice have shown that PGI2 is a potent lipolysis promoter, whereas PGE2 and 15-d-PGJ2 primarily serve anti-lipolytic functions; however, the functions of PGF2α in lipolysis have not been reported [28,29,53]. Some studies have shown that PGE2 and PGF2α remarkably increase macroautophagy (the primary type of autophagy) in mammals [33,54]. However, there is a paucity of research on fish. Interestingly, the present study did not find obvious differences in the contents of major reported functional PGs, such as PGI2, PGE2, and 15-d-PGJ2, although the content of PGF2α in cells increased in response to serum starvation. We further compared autophagy-related features after the addition of exogenous PGF2α and PGE2 to ZFL cells, and observed that PGF2α, but not PGE2, remarkably increased the characteristics of autophagy in these cells (Figure 4), further leading to lipid depletion. Therefore, PGF2α may be a key COX-mediated metabolite involved in starvation-induced lipid degradation in ZFL cells. PGF2α primarily binds to the cell membrane receptor FP. Conversely, some studies have shown that PGs also bind to nuclear receptors, such as members of the PPAR family [55]. Thus, whether exogenous PGF2α promotes autophagy by binding to the membrane or nuclear receptor remains unknown, and the mechanism of PGF2α transport remains unclear. In the present study, PGE2 showed a weaker ability to induce autophagy than the control. A study on mammals showed that PGE2 inhibited lipolysis at concentrations below 10 mM but promoted lipolysis at higher concentrations [56]. Thus, PGE2 concentration in the autophagy of fish hepatocytes remains to be determined, although it possesses a weaker ability to promote autophagy than PGF2α does.

Fatty acids released from LDs via lipolysis or autophagy/lipophagy are transported to the mitochondria for β-oxidation and energy production [57]. Previous studies in freshwater fish grass carp have shown that dietary ARA increases the expression of genes related to mitochondrial function, and that COX pathway-mediated PGs are involved in this process [26,58]. PGs affect multiple mitochondrial functions in mammals. Specifically, 15d-PGJ2 promotes Ca^2+^-induced mitochondrial swelling and cytochrome c release [59]. PGE2 is involved in the dissipation of mitochondrial membrane potential (Δψm) in interleukin-4-activated macrophages [60]. Interestingly, PGI2 showed a protective effect on mitochondrial function, such as oxidative phosphorylation, in preserved rat livers [61]. Nevertheless, no study has demonstrated the role of PGF2α in mitochondrial performance. In the present study, we demonstrated that PGF2α, rather than PGE2, promotes mitochondrial development in fish cells. PGF2α increased the number of mitochondria (mitochondrial staining and mtDNA copy number), enhanced the expression of genes related to β-oxidation and the mitochondrial respiratory chain, and promoted ATP production. These changes are consistent with the degradation of LDs, which may consume fatty acids released from autophagy and lipolysis. However, the underlying mechanism remains unclear. PGF2α likely acts synergistically with cAMP to increase glucose transport, possibly through a PKC-dependent mechanism, in 3T3-L1 adipocytes, indirectly promoting energy utilization of this molecule [62].

The present study had some limitations. First, the interaction of LDs and autophagosomes/lysosomes, as well as autophagy flux, were not comprehensively explored; thus, the role of PGF2α in lipophagy warrants further discussion. Second, chaperone-mediated autophagy (CMA) also plays an essential role in lipid metabolism in fish [63], and we found that the expression of *LAMP2* (a CAM marker gene) was upregulated in response to starvation and PGF2α treatment. Thus, the role of CMA in lipid degradation must be further evaluated.

## 5. Conclusions

**Figure 6:**
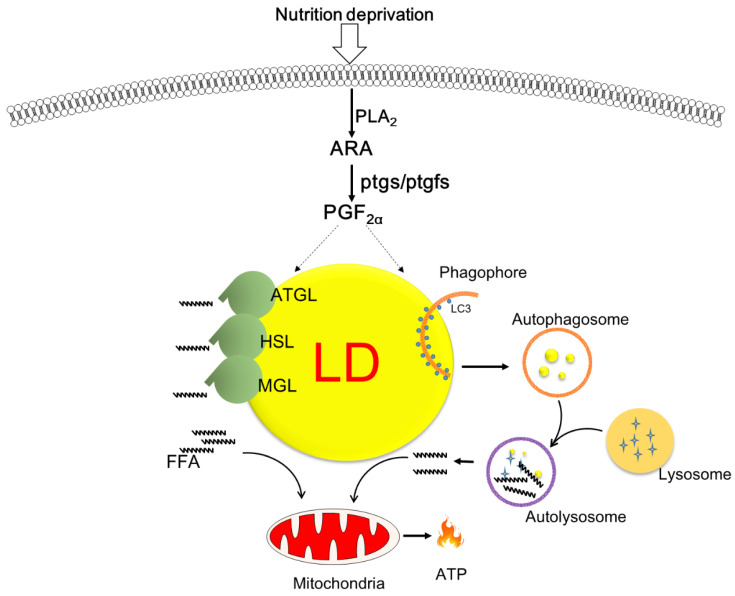
Schematic overview of the proposed role of cyclooxygenase-mediated lipid droplet degradation in response to nutrition deprivation in zebrafish hepatocytes. Serum starvation triggers the release of arachidonic acid and augments the production of PGF2α, which is a key molecule promoting lipid mobilization via autophagy/lipophagy and lipolysis as well as mitochondrial energy production.

The present study provides novel insights into the dynamics of ARA-derived COX metabolites in response to nutritional deprivation in fish hepatocytes. We demonstrated that PGF2α expression was increased and was accompanied by autophagy and lipolysis. Exogenous treatment with PGF2α, but not PGE2, accelerated lipid degradation through enhanced autophagy and lipolysis and improved mitochondrial β-oxidation and energy production (Figure 6). Further studies are warranted to explore the mechanism of action of PGF2α in LD degradation and its role in autophagic flux.

## Data Availability

All the data presented in this study are included in this article.

## Supplementary Materials

The following supporting information can be downloaded at: https://www.mdpi.com/article/10.3390/cells11121870/s1, Figure S1: Effects of knockdown of ATG7 and adipose tissue lipase (ATGL) on the lipid droplet accumulation and lipolysis in zebrafish liver cells under starvation treatment; Figure S2: Effects of knockdown of prostaglandin F synthase (PTGFS) on the PGF2α production in ZFL cells under starvation treatment, Table S1: Primers used in real-time quantitative PCR.
