# Supplementary material for: Prostaglandin 2α Promotes Autophagy and Mitochondrial Energy Production in Fish Hepatocytes
Source: Cells. 2022 Jun 9;11(12):1870. doi: 10.3390/cells11121870 (PMC9220818; doi:10.3390/cells11121870)
Supplement: Supplementary file 1 [file cells-11-01870-s001.zip › cells-1711322-supplementary.pdf]

Table S1. Primers used in real-time quantitative PCR.

Target genes	Forward (5'-3')	Reverse (5'-3')	GenBank NO.
**Atg12**	GCCATCTCACGCTTCCTCA	GCCGTCACTTCCGAAACACT	NM_001246200.1
**Ef1α**	CCCCTGGACACAGAGACTTCATC	ATACCAGCCTCAAACTCACCGAC	NM_131263.1
**Lamp2**	CTCCTCCGACGACTCCTTCT	GGCTGGCGTTCCCACTTA	XM_009291116.3
**Lc3b**	CAGCGGGTGGAGGATGTA	AACGTGGTCAGGAACTAGAAAC	NM_199604.1
**Pparα**	TCAGCGGGAAAGAGGAACAC	GAAACACACGTGCTTTGGCT	NM_001161333.1
**Atgl**	ACACACTTACACCGCGTGAT	AGCACGTTTTCTCCATCCGT	XM_005174256.4
**Hsl**	GCCCAAGGAGCAACAAACTG	TGCAGAGGCTGTTGATGAGG	NM_001316725.1
**Cpt-1**	TCTACCTGAGAGGTCGTGGG	TGACGTTTCCTGCTCTTGCT	NM_001044854.1
**Ptgs1**	AAGGCGGAACTTCAGACTGG	TCCGTGACCCAGACCTTTTG	NM_153656.2
**Ptgs2a**	AAAAGGGACCAGCCTTCACC	TGTTGAACCTCCAGCGTCTC	NM_153657.1
**Ptgs2b**	CTGTGGGTCATGAGGCCTTT	TGTCGCACACTCGGTTATGT	NM_001025504.2
**Ptges**	TGGGTCGAGTTCTTCACAGC	GGCACCTGAGCGATGACATA	NM_001014828.2
**Ptgds**	TGACGCCAAGTTTGACGAGT	CGACCATTTCTGGAGTGCGA	NM_213634.1
**Ptgis**	AACCTCCGCCTGCTTATGAC	GCGCCGAACACTGTCAGATA	NM_001111160.1
**ptgfs**	CCAGATTCTCGATCCGTGGG	CTGCAAAGTCTCAACGCCAC	NM_001185071.1
**Acadm**	ACCCGGGCTGTTAAGAAAGG	GCCAGCAGGAAGTACCAGTT	NM_213010.2
**Acadvl**	GAGGAGTCTCCAGTGGTCCT	GACGCGCACGTTCTCAAAAT	NM_212611.1
**Acads**	AGAGAGTGGTGATGGCGTTG	AATCTCGACACGTCTGCCTC	NM_001003743.1
**Hadhaa**	AACCAGGATGCTTCATCGCA	AACATCTTCTGTCCCGCCTG	NM_001105276.1
**Hadhab**	TTCTGTCCAACCTAACGGGC	GGGGAATCACTGCTTCGACT	NM_001089437.1
**Hadhb**	TTTGATCGCTGCTGGTCAGT	CCTCATTTTGCGGCTGTGTC	NM_200019.1
**Mt-nd1**	CCCACGATTCCGATACGACC	GTGCGATTGGTAGGGCGATA	NC_002333.2
**Sdha**	TGGAGCGTTATGCACCCAAT	TCTTTATCCGGCCCAACACC	NC_007130.7
**Cyc1**	ACGGGCCAGTATGTCATTCG	CAGATCTGAGGCCTTGACCG	NM_001037393.2
**Mt-co1**	CCAGTGTTAGCTGCCGGAAT	GACTTCTGGGTGGCCAAAGA	NC_002333.2
**Mt-atp**	TGCAGTACCCCTATGACTTGC	ATGGGATGGGGGTTCCTTCT	NC_002333.2
**MT-cyb**	TATTCACATCGCCCGAGGAC	GGAAGGACGTAGCCCACAAA	NC_002333.2
**β-actin**	TCTGGTGATGGTGTGACCCA	GGTGAAGCTGTAGCCACGCT	NM_131031.2

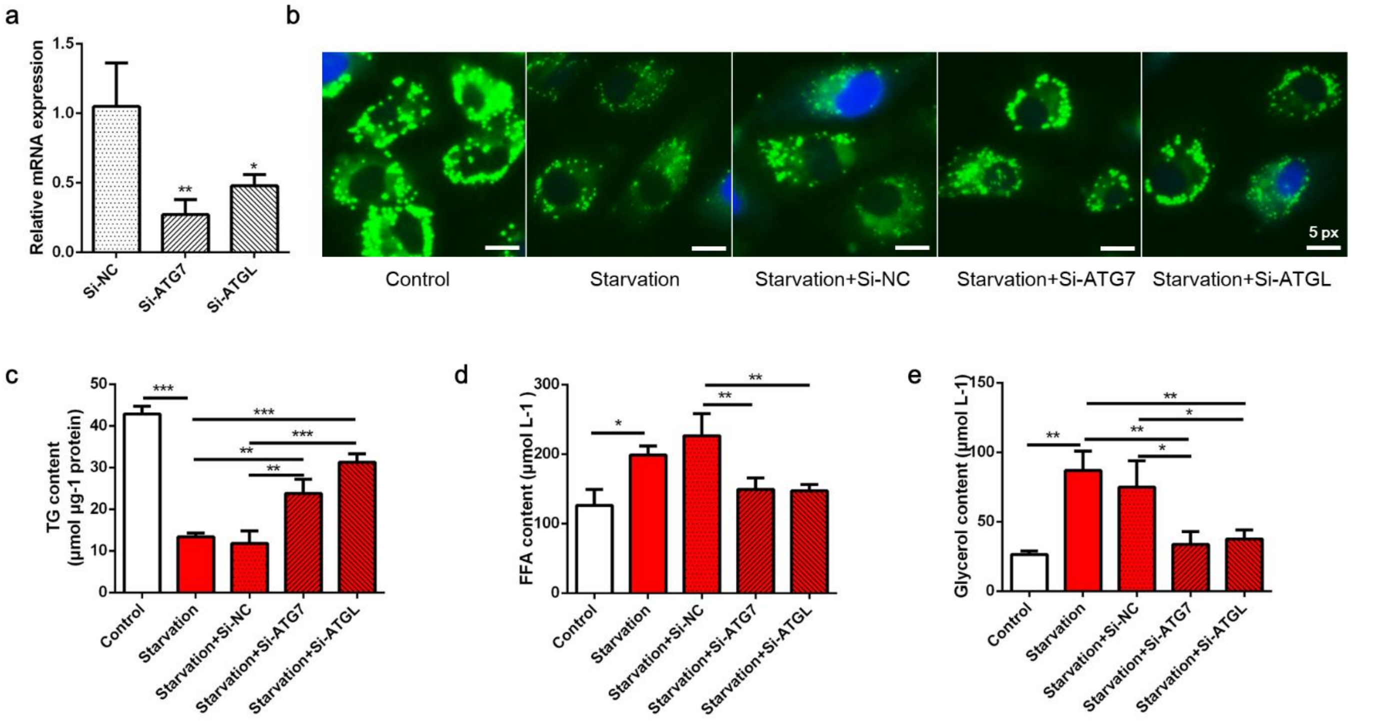

Figure S1. Effects of knockdown of ATG7 and adipose tissue lipase (ATGL) on the lipid droplet accumulation and lipolysis in zebrafish liver cells under starvation treatment. (**a**) Verification of the interference efficiency of ATG7 and ATGL by RNAi technology using qRT-PCR (n = 3). (**b**–**e**) Cells were pre-treated with negative control (NC), ATG7, and ATGL SiRNA and serum starved for 24h. Lipid droplets were stained by bodipy (green), nuclei were stained with DAPI (red). Triglyceride (TG) content in the cells (c), as well as the free fatty acid (FFA; d) and glycerol content in the medium were analyzed (e). (n = 4). Statistical significance is denoted with asterisks as follows: *$p < 0.05$; **$p < 0.01$; ***$p < 0.001$.

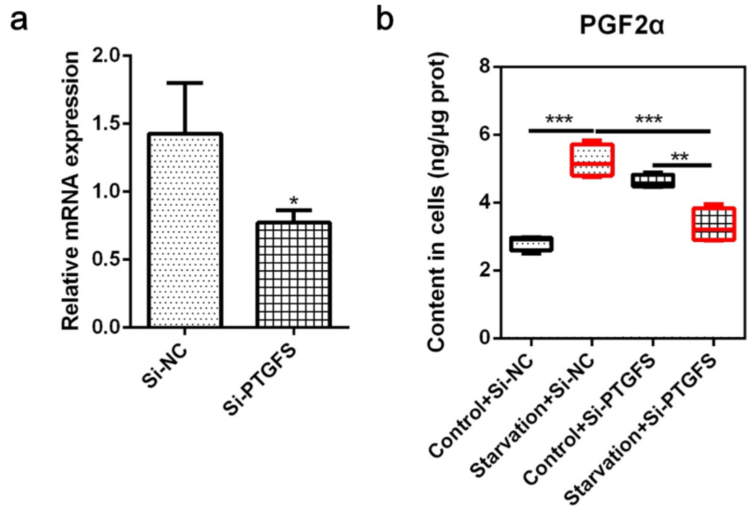

Figure S2. Effects of knockdown of prostaglandin F synthase (PTGFS) on the PGF2α production in ZFL cells under starvation treatment. (**a**) Verification of the interference efficiency of PEGFS by RNAi technology using qRT-PCR (n = 3). (**b**) Cells were pre-treated with negative control (NC), PEGFS SiRNA and serum starved for 24h, the PGF2α content were tested by ELISA assay kits (n = 6). Statistical significance is denoted with asterisks as follows: *$p < 0.05$; **$p < 0.01$; ***$p < 0.001$.
